# Health systems and policy research needed to strengthen the rehabilitation workforce

**DOI:** 10.2471/BLT.22.289032

**Published:** 2022-10-03

**Authors:** Jim Campbell, Jody-Anne Mills

**Affiliations:** a Health Workforce Department, World Health Organization, Geneva 27, Switzerland.; b Department of Noncommunicable Diseases, World Health Organization, Avenue Appia 20, 1211 Geneva 27, Switzerland.

The development of rehabilitation occupations (that is, status of the workforce, number of paid jobs, quality of education and level of regulation, among others) lags far behind others in the health workforce in many, if not most countries in the world.^1^ The labour market failures experienced by the rehabilitation workforce are not unique, but are particularly profound, especially in low- and middle-income countries; they include inequitable distribution, inadequate quality, and paradoxically, both shortages and unemployment due to poor coordination and funding.^2^^,^^3^ While we generally understand how rehabilitation workforce challenges play out and can speculate as to their underlying causes, health policy and systems research is needed to understand what and how solutions can be implemented in different contexts. Here we present several reasons why the rehabilitation workforce has been largely neglected in health system strengthening efforts to date and suggest three health policy and systems research questions that need to be explored to inform policy actions.

## Underlying factors

The scale of discrepancy between the general state of rehabilitation occupations, for instance compared to doctors, nurses and pharmacists,^4^ points to several underlying issues. These issues do not occur in isolation but interact with each other as well as with broader health system challenges. We identify three notable issues.

First, the primary outcome of concern to the rehabilitation workforce is optimal functioning of the patient, but policy-makers have not highly valued functioning in health policy and systems to date. Functioning is critical to obtaining health as defined by the World Health Organization, “a state of complete physical, mental and social well-being and not merely the absence of disease or infirmity.”^5^ However, the number of rehabilitation workforce jobs funded in the public health system and investment made in the education and training of rehabilitation professionals^4^ do not reflect this comprehensive approach to health. Rather, the composition of the health workforce in many countries reflects the legacy of rehabilitation being seen as a luxury service rather than a health service that is essential to the achievement of universal health coverage. The implications of a poorly valued and underinvested rehabilitation workforce are significant, but often inadequately captured. These implications tend to be experienced by people and their families at home and are not observed in health facilities nor included in health data. For example, without an adequate rehabilitation workforce, a person with a spinal cord injury is likely to be discharged from hospital prematurely, with a wheelchair that does not fit their body, to a home that they cannot access. This person will inevitably develop pressure sores or a urinary tract infection soon after discharge, leading to their readmission to hospital or death after a stretch of unnecessary pain.^6^^,^^7^ Similar cases of confinement to homes, health complications and missed opportunities for participation in education, work and meaningful life roles can be found in the context of children with cerebral palsy or autism, people who have had a stroke or a limb amputated, or those with neurodegenerative conditions, among many others.

Second, the development of the rehabilitation workforce is hindered by the weakness of rehabilitation in health systems in general. Many countries experience various limitations in rehabilitation leadership and governance, financing, data, assistive technology, equipment and infrastructure, and services. These limitations contribute to the scope and complexity of workforce challenges because health systems are dynamic and interconnected, meaning that deficiencies in one area (such as leadership or data) will affect other areas. Therefore, even targeted approaches to workforce strengthening must acknowledge and consider the broader health system context. The performance of the rehabilitation workforce is greatly affected by how well it is enabled by the system in which it operates. Even the most well educated and trained workforce will struggle when not incentivized, supported, valued or adequately equipped.

Third, the multiplicity of occupations encompassed within the rehabilitation workforce presents a challenge for advocacy. Independent efforts to communicate the need for and contribution of each rehabilitation occupation may overwhelm ministries of health and education. More than six different rehabilitation occupations exist (Box 1), but many are not well understood by policy-makers. A unified concept of a rehabilitation workforce has the potential to simplify messaging and strengthen advocacy, yet it is not yet widely embraced by the rehabilitation community. Obstacles to the adoption of such a concept may be due in part to (i) rehabilitation occupations wishing to protect and promote their independent professional agendas; (ii) occupations not being aware of or feeling a part of a collective rehabilitation workforce; or (iii) a lack of platforms or opportunities for collective advocacy.

Box 1 **Who is the rehabilitation workforce?**

The rehabilitation workforce is not strictly defined; variations exist in how it is interpreted and composed in different countries and contexts. However, rehabilitation is characterized by a focus on functioning, with rehabilitation workers delivering interventions that help people with health conditions perform to optimal levels in their daily lives. These interventions may address challenges in mobility, communication, cognition or mental health that are limiting the independence or participation of people with health conditions.^8^

Rehabilitation interventions are delivered by a diverse range of occupations, such as audiologists, occupational therapists, physiotherapists, physical and rehabilitation medicine doctors, prosthetists and orthotists, and speech and language therapists, among others.^9^ Rehabilitation occupations are typically supported by assistants and/or technicians, the qualifications and scope of practice for which differ greatly from country to country.

Bringing the rehabilitation workforce in line with other health occupations requires coordinated actions across sectors, including health, education, labour and finance. For strategies to make a meaningful impact on the rehabilitation workforce, they need to be evidence informed. Here we point to three health policy and systems research questions that can guide and accelerate efforts towards strengthening the rehabilitation workforce.

## Contribution of research

Health policy and systems research focuses on what works, for whom and under what conditions.^10^ Such research is valuable in the development and analysis of health policy, but also in contextualizing policy solutions. In view of the underlying challenges detailed above, the following health policy and systems research questions warrant exploration.

First, what factors influence how rehabilitation workers are understood and valued by the broader health workforce, the public and policy-makers in different contexts? This question is critical to addressing all challenges, but particularly that of under prioritization. How the rehabilitation workforce is understood and interpreted may vary considerably. For example, in some contexts the workforce may be strongly associated with the disability agenda and perceived to be the responsibility of social affairs, while other countries will consider it to be a core component of the health workforce. Furthermore, what policy-makers value and subsequently prioritize is shaped by many factors, including population needs, resource availability, historical priorities and many others.

Second, how can the rehabilitation workforce be optimized to expand access to essential rehabilitation interventions? This question seeks information to better understand the composition, organization and operation of rehabilitation and other health occupations to deliver rehabilitation. These factors are likely to be highly context specific and affect workforce production and employment, as well as task sharing. This question is a critical health policy and systems research question, as it responds to the situation of rehabilitation workforce in many contexts (including at the subnational level), whereby a health system must make the most efficient use of a very limited pool of workers.

Third, how can the public and private sectors work together to address labour market failures of the rehabilitation workforce? The private sector has tremendous potential to both cause and address labour market failures through influencing the production and absorption of workers.^11^ The private sector may have a particularly powerful role in countries that do not yet have the potential to develop and support the rehabilitation workforce needed by the population. However, public–private partnerships are complex, and affected by the regulatory environment and the market. A better understanding of the relationship between the public and private sector in relation to the rehabilitation workforce may help guide constructive partnerships that could help fill critical gaps in the health system.

We need to address the scarcity of health policy and systems research relevant to the rehabilitation workforce. The questions posed above are by no means exhaustive but represent areas of knowledge that have the potential to accelerate the development of the rehabilitation workforce and subsequently strengthen health systems. The information gained through this research will serve to deepen our understanding of the problem, but also contribute to the implementation of context-specific solutions.
